# Imaging analysis of the malignant transformation of bile duct hamartomas in the liver: A case report and literature review

**DOI:** 10.3892/etm.2022.11358

**Published:** 2022-05-09

**Authors:** Yuan Yuan, Gang Fu, Feng Wan, Xu-Lei Chen, Jun Feng

**Affiliations:** 1 Department of Ultrasound, The Second Hospital of Wuxi Affiliated to Nanjing Medical University, Wuxi, Jiangsu 214002, P.R. China; 2 Department of Pathology, The Second Hospital of Wuxi Affiliated to Nanjing Medical University, Wuxi, Jiangsu 214002, P.R. China

**Keywords:** bile duct hamartoma in the liver, malignant transformation, contrast-enhanced ultrasonography, enhanced computed tomography, imaging analysis

## Abstract

Bile duct hamartoma in the liver (LBDH) is relatively rare among the hepatic space-occupying lesions that occur in adults, and the malignant transformation of LBDH is even rarer. In the present case report, a 63-year-old male was found to have two space-occupying lesions in the right lobe of the liver upon ultrasound examination. Enhanced computed tomography (CT) suggested benign hepatic haemangioma, and contrast-enhanced ultrasonography (CEUS) suggested well-differentiated hepatocellular carcinoma. The final pathology results revealed the malignant transformation of LBDH into well-differentiated intrahepatic cholangiocarcinoma. Improved recognition of this type of rare disease can be obtained by radiographic analysis of this case. These findings contribute to a better understanding of the enhanced development pattern of this disease on contrast-enhanced CT, as well as on CEUS.

## Introduction

Bile duct hamartoma in the liver (LBDH), also known as von Mayenburg complex (VMC) and polycystic bile duct hamartoma, is a benign malformation of the intrahepatic bile duct associated with bile duct plate defects (1,2). The clinical symptoms and signs of disease in patients are often atypical, so these tumours are usually incidentally found during physical examination, exploratory laparotomy or autopsy. Some cases have mild pain in the right upper abdomen, and the blood α-fetoprotein (AFP) and carcinoembryonic antigen (CEA) levels are generally normal (3). LBDH is considered to be caused by abnormal development of intrahepatic bile ducts. Histologically, it consists of inflammatory cells, bile ducts and fibrosis. Macroscopically, LBDH appears as multiple small greyish-white nodules scattered below the liver capsule and around the portal vein. These lesions usually do not communicate with the bile duct tree. Microscopically, the bile duct consists of a series of dilated branching cystic bile ducts lined with a single cuboid epithelial cell layer surrounded by a rich fibrocollagen matrix. The diameter of each lesion is 0.1-1.5 cm. The lumen of the bile duct usually contains bile-stained granular matter (4). LBDH is extremely rare, and its incidence at autopsy is 0.6-5.6% (5). At the same time, due to the lack of an adequate understanding of LBDH ultrasonic manifestations, misdiagnosis easily occurs. The ultrasound misdiagnosis rate of this disease is high at ~80%. LBDH must mainly be differentiated from the following diseases: Liver cysts, chronic liver disease, cirrhosis, diffuse liver parenchymal lesions, Caroli disease, multiple hepatic hemangiomas and multiple intrahepatic metastases (6); therefore, investigations into how to effectively improve the imaging diagnosis level of LBDHs are warranted. At the same time, LBDH has a certain tendency toward malignant transformation, and the clinical and imaging manifestations lack specificity. By comparing the differences in imaging manifestations on enhanced computed tomography (CT) and contrast-enhanced ultrasonography (CEUS), the imaging diagnostic performance can be improved to a great extent.

## Case report

### Patient

An elderly male, aged 63 years, was found to have space-occupying lesions ~2 cm in size in the liver during a routine physical examination at an external hospital. Therefore, the patient was admitted to The Second Hospital of Wuxi Affiliated to Nanjing Medical University (Wuxi, China) in January 2021. The patient was generally in good condition without obvious symptoms of discomfort and no yellow staining of the skin or sclera. The patient had a flat and soft abdomen, and no abnormal masses could be palpated. Laboratory examination revealed that the tumour marker levels, including that of AFP, CEA, carbohydrate antigen (CA)-125 and CA-199, were within the normal ranges (data not shown). The results for hepatitis virus markers were negative.

### Ultrasound

The patient was subjected to a routine liver ultrasound examination. Entire liver sections were scanned with a conventional US. When a target lesion was found, the maximum cross section of the tumour diameter and blood supply were examined and recorded. The ultrasound examination showed diffuse chronic liver disease and cirrhosis. Two space-occupying lesions were found in the right lobe of the liver: One close to the liver capsule and located in segment six (S6), and the other 1.5 cm away from the liver capsule and located in segment five (S5). Both masses were hyperechoic and had uneven internal echoes, unclear boundaries and no obvious capsules. If benign, these masses could be hepatic haemangiomas, while if malignant, they may be hepatocellular carcinoma (HCC). Therefore, the ultrasonographer suggested further examinations to determine the nature of the masses.

### Enhanced CT

The abdomen was scanned by a Toshiba Aquilion ONE64 slice spiral CT scanner. The scanning parameters were 200 mAsec, 120 kV and a slice thickness of 0.5 mm. The contrast agent injected for enhanced scanning was ioversol (320 mg I/ml). The injection flow rate was 3.0 ml/sec and the dose applied was 1.5 ml/kg of body weight. Arterial phase scanning was performed 25 sec after injection, venous phase scanning was performed 60 sec later and delayed phase scanning was performed 120 sec after this.

**Figure 1:**
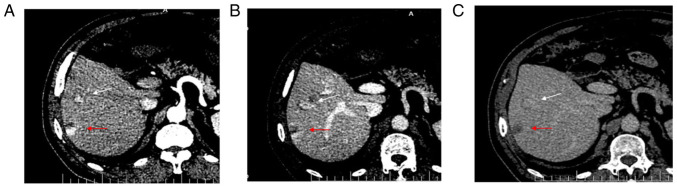
CT of the abdomen. (A) Contrast-enhanced CT showing local enhancement of both the segment 5 (white arrow) and segment 6 (red arrow) lesions in the arterial phase. (B) Enhanced CT showing continuous enhancement of both lesions in the portal vein phase. (C) Enhanced CT showing continued enhancement of the lesion during the delayed period, without significant clearance. Contrast-enhanced CT revealed a benign hepatic haemangioma. CT, computed tomography.

Enhanced CT showed local enhancement in the arterial phase (Fig. 1A) and continuous enhancement in the portal (Fig. 1B) and delayed (Fig. 1C) phases, without obvious clearance. Enhanced CT showed a strong-equal-equal enhancement mode, suggesting a benign hepatic hemangioma. The results of the enhanced CT showed that the mass was a benign hepatic hemangioma.

### CEUS

At the suggestion of the ultrasound doctor, the patient underwent CEUS. CEUS was initiated using a Resona 7 (Shenzhen Mindray Bio-Medical Electronics Co., Ltd.) ultrasound instrument. After a bolus injection of 1.6 ml SonoVue (Bracco Group) through a peripheral venous cannula, a 5-ml saline flush was used and the timer on the sonography was started. Observations were made until the microbubbles cleared from circulation (usually up to 5 min). All video clips were recorded and then transferred to a hard disk.

**Figure 2:**
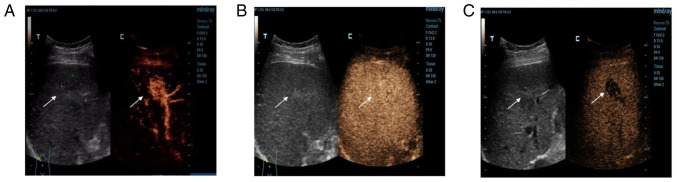
Contrast-enhanced ultrasound examination of liver segment 5 lesion. (A) The development time of the hyperechoic mass (arrow) was significantly earlier than that of the hepatic parenchyma during the arterial phase, and the range of the mass was wider than that of conventional images. (B) The mass (arrow) was continuously observed and developed in the portal vein phase. (C) The mass (arrow) in the delay period was not fully clear until 6 min later.

**Figure 3:**
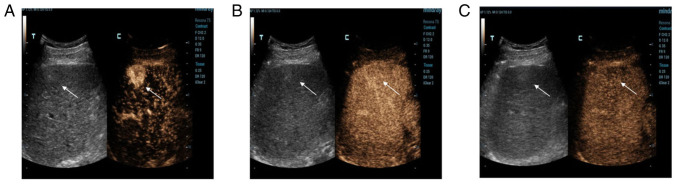
Contrast-enhanced ultrasound examination of liver segment 6 lesion. (A) The lesion (arrow) developed rapidly in the arterial phase. (B) The lesion (arrow) continued to develop in the portal vein stage. (C) In the delayed phase, the lesion (arrow) regressed slowly.

The examination showed a mass in S5 (Fig. 2A). At 13 sec, the hepatic artery began to develop, while the hyperechoic mass developed rapidly, reaching peak intensity at 17 sec, and then the surrounding liver parenchyma began to develop. The development time of the hyperechoic masses in the arterial phase was significantly earlier than that in the hepatic parenchyma, and the range of the mass was wider than that of conventional images. Upon further observation, the enhancement continued to develop (Fig. 2B) and then began to decline 2 min later. The mass was completely cleared after 6 min (Fig. 2C). The other mass in S6 was developed in the same way; it developed rapidly in the arterial phase (Fig. 3A), continuously in the portal phase (Fig. 3B) and slowly decreased in the delayed phase (Fig. 3C). CEUS showed a strong-equal-low development mode, so malignant lesions were first considered.

Next, the patient now presented with liver cirrhosis, which led to the consideration of HCC; however, due to the late and slow regression, a highly differentiated HCC was considered.

Therefore, from the imaging examinations, CEUS had revealed a highly differentiated HCC, enhanced CT showed benign lesions and CEUS showed malignant lesions.

### Surgery

Finally, the patient chose to undergo surgery, and during the operation, the liver exhibited small nodular cirrhosis. The two masses were located by B-ultrasound during the operation and were found to be located at the junction of S5 and S6. The nodular masses were grey in pathological appearance. One was close to the liver capsule (3.0x2.5x2.0 cm in size), and the other was 1.5 cm away from the liver capsule (3.0x2.0x2.0 cm in size). The boundary remained clear.

### Histology

The hepatobiliary surgeon removed two lesions and sent them to the Department of Pathology for examination. Pathologists sectioned and stained the samples, and performed histological and immunohistochemical examinations. First, the specimen was placed in fixative (10% formalin) overnight at a normal atmospheric temperature. The samples were taken on the second day (the sample size was 1.5x1.5x0.2 cm). The samples were then dehydrated overnight in a dehydrator. On the third day, the sample was placed in an embedding box for embedding (65˚C in paraffin). After embedding, they were placed in a paraffin slicer for tissue sectioning at a thickness of 3-4 µm. The cut sample slices were placed on the slides, and the slides were baked on an electric heating plate at 60-70˚C for 1-1.5 h. H&E staining was then performed. The whole H&E staining process consisted of dewaxing, dyeing, dehydration, making the section transparent and sealing. First, wax was removed from slices using xylene three times for 5-10 min each. The xylene was then washed away with alcohol (anhydrous, 95, 80 and 70% sequentially for 1 min). Next, the slices were rinsed with water for 2 min. The slices were then stained with haematoxylin for 5 min. The cells were rinsed with water again for 1-3 min after staining. After the sections were rinsed with water, they were rinsed with acidic alcohol (1%) for 20 sec. The sections were rinsed in water for >15 min until the nuclei turned blue. Eosin solution was used for dyeing for 30 sec to 1 min, and each alcohol concentration (anhydrous, 85 and 95%, twice) was used for 1-2 min/wash. The tissue sections were made transparent three times (2 min/wash) with xylene to ensure the transparency of the sections. Finally, the tablet was sealed with neutral gum and labelled accordingly. Images were then taken under a light microscope (Nikon Corporation) at x200 magnification.

**Figure 4:**
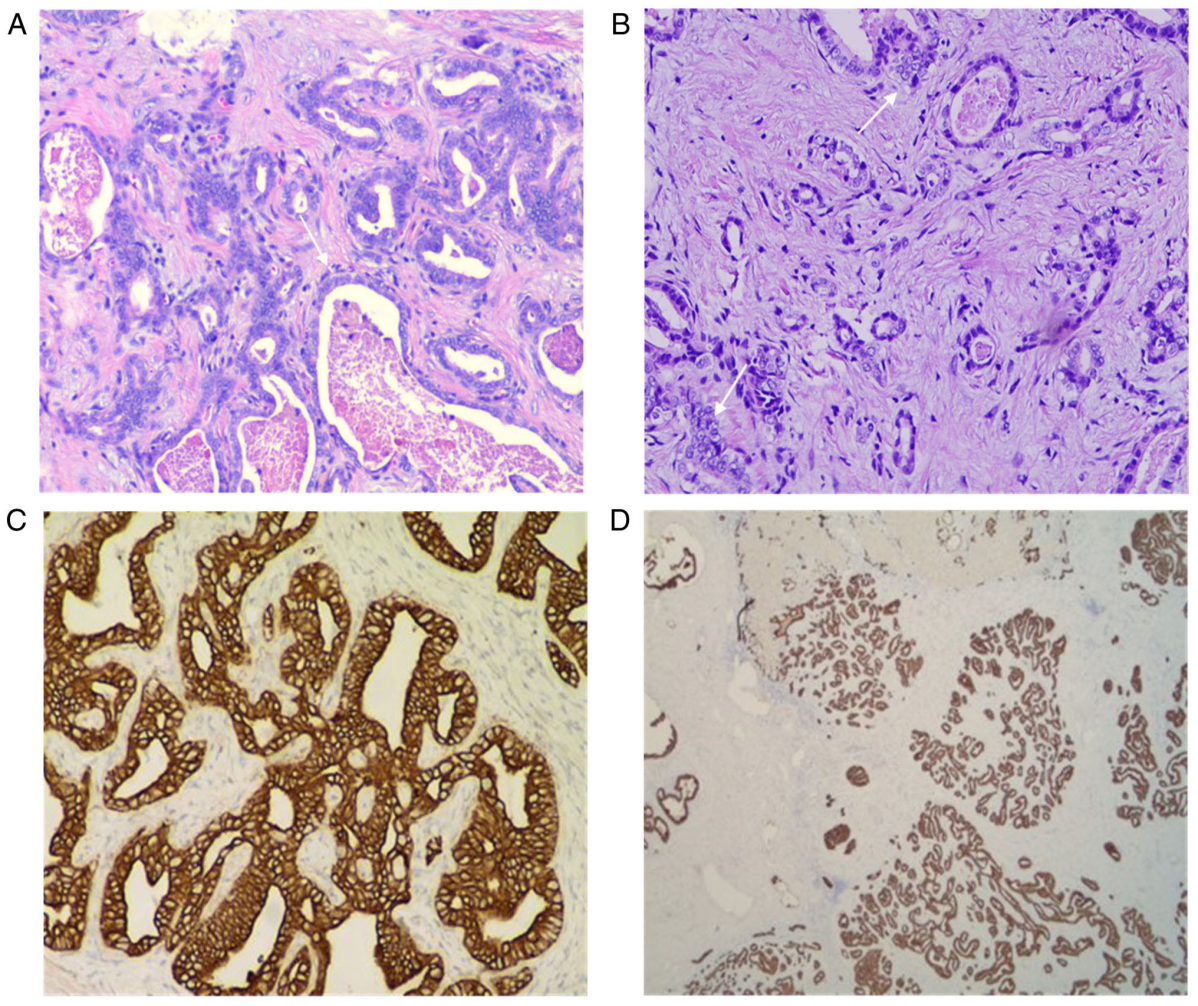
Histological diagnosis of bile duct hamartoma in the liver with malignant transformation into well-differentiated intrahepatic cholangiocarcinoma. (A) Obvious hyperplasia of interlobular bile ducts (arrow) was seen, with lobulated and partly cystic dilatation (H&E, x200 magnification). (B) In some areas, the epithelial cells (arrows) of the bile duct showed agglomeration and were heteromorphic, were arranged in glandular tubular and papillary shape, and the nucleoli were obviously accompanied by fibrous hyperplasia (H&E, x200 magnification). (C) CK8(+) and (D) CK19(+) immunohistochemistry results (x200 magnification). CK, cytokeratin.

Under a light microscope, obvious hyperplasia of the interlobular bile ducts was observed, along with lobulated and partial cystic dilatation, suggesting LBDH (Fig. 4A). In addition, the agglomeration of bile duct epithelial cells was observed, and there were certain areas that had atypia and adenotubular and papillary arrangements, with obvious nucleoli accompanied by fibrous hyperplasia (Fig. 4B).

### Immunohistochemistry

Following tissue preparation as aforementioned, the EliVision^™^ Super system (MXB Biotechnologies) was applied according to the manufacturer's instructions. The system used a two-step method in which the primary antibodies were mouse anti-human IgG monoclonal antibodies [hepatocyte, cat. no. MAB-0249; CD34, cat. no. KIT-0004; cytokeratin (CK) 7, cat. no. KIT-0021; CK20, cat. no. KIT-0025; CK19, cat. no. KIT-0030; Glypican-3, cat. no. KIT-0036; thyroid transcription factor 1 (TTF-1), cat. no. MAB-0599; and CK8, cat. no. KIT-0034; all MXB Biotechnologies]. Then a large amount of secondary antibody (anti-mouse/anti-rabbit) IgG polymer (cat. no. TT-0801; MXB Biotechnologies) was indirectly linked to the antigen-bound primary antibody in the sample by linking with reaction amplifiers.

After dewaxing and hydration, the paraffin sections were washed with water. Pre-treated tissue sections were used (depending on the specific instructions of primary antibody). Peroxidase blocking reagent (3% H_2_O_2_) was dripped onto the sections, and the sections were incubated at normal atmospheric temperature for 10 min and rinsed with PBS (cat. no. PBS-0060) three times (3 min/wash). After rinsing, the primary antibody was added to the sections and incubated at normal atmospheric temperature for 60 min. After incubation, the sections were rinsed with PBS again three times (3 min/wash). Reaction magnifying agent was then added to the slices, which were incubated for 15 min at normal atmospheric temperature. After incubation, the sections were rinsed with PBS again three times (3 min/wash). After rinsing, high-sensitivity enzyme-conjugated anti-mouse/anti-rabbit IgG polymer was added to the sections, which were incubated at room temperature for 15 min. After incubation, the sections were rinsed with PBS again. A total of 120 µl DAB chromogenic reagent (Titan Super) was added to the slices after washing. Finally, the sealing piece was redyed with haematoxylin. Images were then taken under a light microscope (Nikon) (image magnification is 200 times).

**Figure 5:**
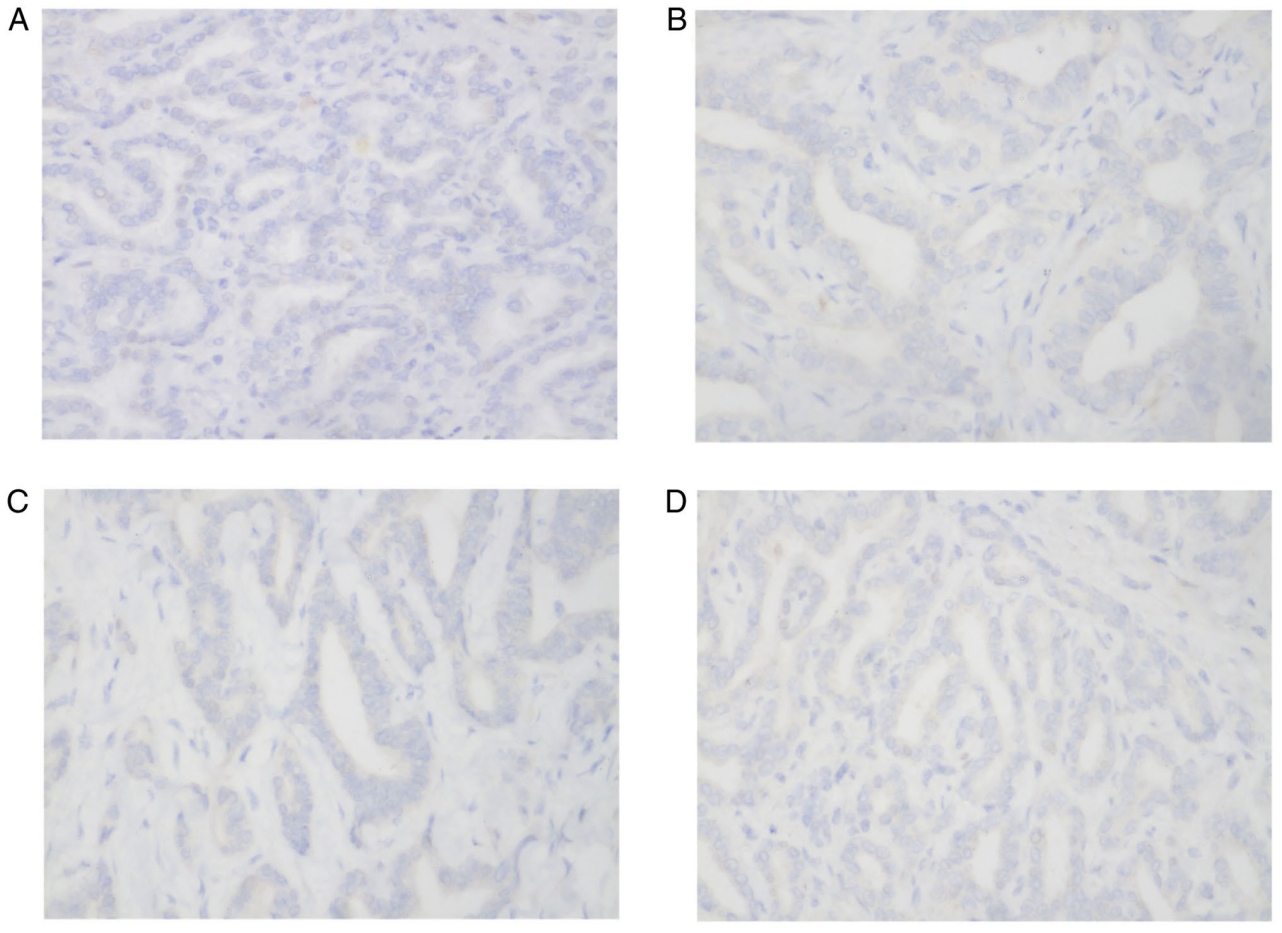
Negative markers of cancerous areas on immunohistochemistry. Cancerous areas were negative for (A) hepatocyte markers, (B) cytokeratin 20 markers, (C) thyroid transcription factor 1 markers and (D) Glypican-3 markers.

The immunohistochemical staining results of the tumour cells from the patient showed the following results: Hepatocyte(-) (Fig. 5A), CD34(-), CK7(+) and CK20(-) (Fig. 5B), CDX2(-), CK19(+), TTF1(-) (Fig. 5C), Glypican-3(-) (Fig. 5D), CK8(+) and S100(-). There was no hepatocyte origin due to the hepatocyte(-) results. CK8 (Fig. 4C) is mainly used to label non-squamous epithelium and can be used for the diagnosis of adenocarcinoma and ductal carcinoma. CK19 (Fig. 4D) is mainly used to label different types of monolayer epithelia, including the glandular epithelium, and is mainly used for the diagnosis of adenocarcinoma. CK19 has no staining for liver cells, and has specific staining for bile duct epithelium and intrahepatic cholangiocarcinoma (ICC), with a positive rate of 77-100%. CK19 is the best immunohistochemical marker for the diagnosis of ICC at present (7). A previous case study (8) reported a diagnosis of bile duct adenoma, with the immunohistochemical analysis revealing CK7(+), CK19(+), CEA(-) and AFP(+) results. In another case report on multicystic biliary hamartoma (9), immunohistochemical staining revealed that dilated ducts were positive for CK19. As indicated, hepatocytes do not express CK19, whereas the present case was CK19(+), suggesting that the tumour originated from the bile duct epithelium, and the final histological diagnosis was of an LBDH malignant transformation into a well-differentiated ICC. The final pathological results of this case were different from those suggested by enhanced CT and CEUS. According to the pathological mechanism of LDBH, the enhanced CT and CEUS manifestations of this rare case were analysed and explained by comparing the pathological principles and imaging principles.

## Literature review

### Search strategy

The PubMed (https://pubmed.ncbi.nlm.nih.gov/), EMBASE (https://www.embase.com/) and Web of Science (http://webofscience.com) databases were systematically searched up to June 2021. The following key words were used: (‘bile duct hamartomas in liver’ OR ‘malignant transformation’) AND (‘contrast-enhanced ultrasonography’ OR ‘enhanced CT’ OR ‘imaging analysis’). Only studies published in English or Chinese and full-text journal articles of original studies were included. All other studies were excluded. Furthermore, the references cited in the relevant studies were reviewed for additional eligible publications. In the process of the literature review, >30 case reports and literature reviews were searched. The majority of studies were for individual cases, and most of the diagnostic methods focused on imaging examinations. Imaging examinations mainly included ultrasound, enhanced CT, MR and CEUS. Among them, the use of CEUS was relatively rare, and mostly reports were on multiple benign lesions. No case report on CEUS manifestations of the malignant transformation of LBDH was found.

## Discussion

LBDH, also known as VMC, was first described by von Meyenburg (10) in 1918. The lesions are usually small (<5 mm) and appear as multiple scattered lesions throughout the liver. The aetiology is not yet clear. Some scholars believe that LBDH is caused by the abnormal development of the bile duct plate or the abnormal remodelling of the bile duct plate during embryonic development (11-14). From the 6 to 8th weeks of embryonic development, the hepatocytes in contact with the mesenchyme around the portal vein express bile duct keratin, which induces the hepatocytes near the portal vein branch to differentiate into bile duct epithelial cells. These cells form a double-layer cuff-like bile duct plate around the portal vein branch that contains the capillary plexus, which forms the hepatic sinus system. After the 12th week of embryonic development, a more mature bile duct is formed around the hepatic portal vein, the excess bile duct becomes apoptotic, and finally, a bile duct network is formed around the portal vein. The order of bile duct reconstruction is from the hilar to the peripheral part of the liver, first forming the bold duct, then the segmental bile duct, the interlobular bile duct and, finally, the smallest capillary bile duct. Under normal circumstances, any excess bile duct will be degenerated and absorbed. When the transformation from the bile duct plate to the bile duct in late embryos is blocked and the absorption is insufficient, a labyrinthine bile duct is formed, resulting in the retention of secretory epithelial cells and fluid in the surrounding tissue, which develops into cystic lesions (15). Moreover, due to its high incidence with visceral polycystic lesions, it has been inferred that this cystic formation has a certain genetic tendency (16), and some scholars have speculated that it is the result of liver inflammation, ischaemia or genetic abnormalities (17).

LBDHs have a variety of ultrasonic imaging features, manifesting as multiple divergent cystic echoes in the liver, and diffuse echo changes in the liver parenchyma, with patchy high echo and low echo, and intrahepatic multiple comet tail signs. The different manifestations on ultrasound images are closely related to the size of the dilated bile duct structure (18). A cystic dilated bile duct wall is composed of bile duct epithelial cells, ductal glands and fibrous connective tissue surrounded by a fibrous matrix tissue (19). When the dilated bile duct is visible, it shows a cystic echo pattern. There is a high concentration of cholestasis in the dilated bile duct lumen, so there is no enhancement effect behind some of the cystic lesions. When the dilated bile duct is small, it is difficult for ultrasound to depict the internal anechoic part, and only the thick capsule wall interface can be observed, so ultrasound shows only hyperechoic or hypoechoic nodules. The appearance of comet tail signs is often caused by multiple reflections off the dilated bile duct. If carefully observed, the front of the comet tail is often accompanied by hyperechoic or hypoechoic lesions. Occasionally, aggregated bile duct hamartomas can also appear as large solitary lesions on imaging (20).

A small number of patients experience malignant progression of multiple LBDH to ICC (more common) or HCC. The risk factors for malignant transformation include chemical or mechanical stimulation, cholestasis or chronic inflammation caused by calculi (21-23). Therefore, long-term follow-up of patients with clearly diagnosed multiple LBDHs is important. If there is a definite malignant manifestation, the lesion can be removed in time, and the prognosis of the patient can be greatly improved.

In a number of cases of LBDH reported in the past, ultrasound, CT, magnetic resonance imaging (MRI), digital subtraction angiography (DSA) and magnetic resonance cholangiography (MRC) have been used to help diagnose the disease (24,25). There are few reports in the existing literature on CEUS manifestations of isolated or sporadic LBDH, and all of these reports are of benign disease, while CEUS manifestations of malignant LBDH are almost completely absent from the literature. CEUS and enhanced CT are certainly first-line tests for such focal liver lesions, and in most cases, they provide sufficient diagnostic information. If the results of ultrasound and CT are unclear or the possibility of a rare disease is considered, the clinician may recommend subsequent MRI. LBDH is a benign hepatic cystic lesion that may undergo cystic enlargement and internal haemorrhage. Complicated giant-LBDH coexists with smaller LBDH and the MRI features of giant-LBDH are characteristic (26). MRI is considered to be the gold standard for the diagnosis of LBDH due to its higher sensitivity and specificity than CT. However, as the current case was very rare, presenting two space-occupying lesions and not the typical multiple and variously sized cystic lesions, the clinician did not consider MRI first when selecting the type of imaging examination. It is also necessary to consider whether the physical condition of the patient is suitable for MRI examination and the economic burden on the patient, as an MRI examination is relatively expensive. In addition, through a review of the literature, it was found that such isolated or sporadic bile duct hamartomas reported in numerous studies were mostly examined only by ultrasound and CT before diagnosis, and rarely by MRI (27). Moreover, as an invasive interventional examination, DSA has great advantages for the diagnosis of liver space-occupying lesions. However, with the continuous promotion of minimally invasive examinations and even non-invasive examinations, its current application is mainly focused on the heart, brain and other organs, while its application in liver tumours is gradually decreasing. Therefore, DSA examination is not the first choice in this case. MRC is mainly applied in patients with bile duct dilation and suspected biliary tract lesions (28). No obvious intrahepatic biliary tract changes were observed in the present patient case, and the disease mainly manifested as focal space-occupying lesions. Therefore, CEUS and enhanced CT, which can be performed in primary hospitals, were selected for imaging comparison.

In the imaging analysis of this case, enhanced CT showed a strong-equal-equal enhancement mode, suggesting a benign hepatic haemangioma. However, CEUS showed a strong-equal-low development mode, so malignant lesions were first considered. Next, the patient presented with liver cirrhosis, which led to the consideration of HCC first, but due to the late and slow regression, a less well-differentiated HCC was then considered. Therefore, enhanced CT and CEUS were performed, but one suggested a benign lesion, and the other suggested a malignant lesion. The question thus becomes why such discrepant findings were observed. Ultrasound contrast agent is a blood pool contrast agent that is injected into the human body through a peripheral vein and enters the hepatic mass through the pulmonary circulation to achieve enhancement and development (29). Due to the obvious differences in the vascular distribution and haemodynamic characteristics between benign and malignant liver tumours, contrast agents show different development patterns in the liver, which has become an important basis for differentiating between benign and malignant tumours. As malignant liver tumours are rich in blood vessels and as their blood supply is from the hepatic artery, their arteries dilate, have circuity, and are around and at the centre of an abnormal proliferation of tumour blood vessels and arteriovenous anastomosis, with ~75% of the normal liver parenchyma being supplied by the portal vein (30). The contrast agent enters the tumour early but fades fast, so the enhancement time for this is short, while it enters the liver parenchyma late, so the enhancement time for this is long. Therefore, the vast majority of enhancement modes for enhanced CT and CEUS of malignant liver tumours are similar, showing rapid enhancement and rapid decline (fast in and fast out) (31). However, contrast-enhanced CT uses an ionic contrast agent that can penetrate into the mass organization before clearance, so for some non-hepatocellular carcinoma tumours, such as intrahepatic bile duct carcinoma (ICC), the contrast agent will gradually penetrate into the tumour; thus, these tumours are characterized by rapid enhancement without decreasing the enhanced mode (fast in and slow out). This enhancement model is a double-edged sword that can be used to distinguish HCC from ICC, but can easily lead to a misdiagnosis of a benign liver tumour such as hepatic haemangioma. This occurs since hepatic haemangioma is composed of sinuses of different sizes, and its blood flow velocity is relatively slow compared with that of HCC. Therefore, the contrast agent does not easily enter and exit, thus presenting a typical image of slow enhancement and slow decline (slow in and slow out). The tumour in the present case was an LBDH that turned into a highly differentiated ICC, which showed a pattern of continuous enhancement without regression in the delayed stage, so it could be easily misdiagnosed as a benign haemangioma. The ultrasound contrast agent is a real blood pool contrast agent that will not enter the tissue space, so the enhancement mode will be fast enhancement and fast decline (fast in and fast out) for the vast majority of malignant tumours. For the differentiation between benign and malignant tumours, CEUS has obvious advantages over CT, as it can accurately reflect the characteristics of blood flow distribution and the haemodynamic changes in liver tumours, which is why CEUS could be used to diagnose the malignant tumours in the present case. However, for the differentiation of different pathological types of malignant lesions, such as HCC and ICC, enhanced CT may be more intuitive than CEUS imaging, as the contrast agent can better penetrate into the tissue space.

In addition, enhanced CT has time constraints. For some masses with late regression, imaging technicians are likely to fail to capture the point of regression during the delay period, resulting in the illusion that the mass does not fade during the delay period and thus causing the mass to be misdiagnosed as benign. Moreover, enhanced CT exposes patients to a certain level of radiation, so it cannot be applied for a long time. CEUS, by contrast, is full-course, real-time and dynamic, and can be applied for as long as desired, providing a clearer indication of whether the delay has subsided. In conclusion, enhanced CT and CEUS both have their advantages. CEUS is more advantageous in differentiating benign from malignant masses, while enhanced CT is more intuitive and accurate in differentiating malignant tumours of different pathological types. In the present case, when a benign haemangioma was indicated by enhanced CT due to the strong-equal-equal enhancement mode, CEUS assisted in the diagnosis of malignancy due to the strong-equal-low enhancement mode, both of which provided different diagnostic ideas and surgical bases for the clinic, helped clinicians remove the lesion early, and greatly improved the prognosis of the patient. The advantages of CEUS in differentiating benign from malignant lesions should be emphasized.

The time it takes for the contrast agent to regress in the delayed stage of HCC can reflect the blood supply ratio of arteries and portal veins, which can be used to judge the level of differentiation of HCC. As the degree of malignancy of HCC increases, the contrast agent regression time decreases (32,33). The question remains as to whether the development pattern is similar for cases of ICC. The pathology results of the present case revealed that the lesion was a highly differentiated ICC, which was illustrated by late regression on CEUS, consistent with the angiographic pattern of HCC. However, due to the rarity of this disease, a large amount of data and case accumulation are still needed to prove this link.

There are few reports in the existing literature on CEUS manifestations of isolated or sporadic LBDH, and all of these reports are of benign disease. Meanwhile, CEUS manifestations of malignant LBDH are almost completely absent from the literature (34). In the present case, the manifestations of malignant LBDH on CEUS were significantly different from those of benign LBDH.

The differences between enhanced CT and CEUS in the current rare case were analysed, and the imaging principles and pathophysiological basis of the two examinations in the diagnosis of LBDH malignant transformation were analysed, providing new diagnostic ideas and examination methods for subsequent clinical work.

The present study reveals that the use of a combination of multiple imaging methods in the diagnosis of this disease can greatly improve the rate of clinical diagnosis and reduce the rate of misdiagnosis, and reveal any malignant trend in the lesions in a timely manner, thus helping clinicians remove the lesions as early as possible and greatly improving the prognosis of the patient.

In conclusion, LBDH is a rare lesion with malignant potential that lacks specific clinical and imaging manifestations. By analysing the characteristic imaging features of intrahepatic bile duct hamartomas that correspond to its pathological features, combined with the imaging rules of enhanced CT and CEUS, the diagnostic accuracy of imaging can be improved to a great extent. Malignant lesions can be found early, the lesion can be removed in a timely manner and the prognosis of these patients can be greatly improved.

## Data Availability

All data generated or analysed during this study are included in this published article.
